# Reliability and diagnostic validity of a joint vibration analysis device

**DOI:** 10.1186/s12903-017-0346-9

**Published:** 2017-02-16

**Authors:** Sonia Sharma, Heidi C. Crow, Krishnan Kartha, W. D. McCall, Yoly M. Gonzalez

**Affiliations:** Department of Oral Diagnostic Sciences, School of Dental Medicine, University at Buffalo, Buffalo, NY 14214 USA

**Keywords:** Joint vibration, Temporomandibular disorders, Reliability, Diagnostic validity, Factor analysis

## Abstract

**Background:**

This observational study was designed to evaluate the reliability and diagnostic validity of Joint Vibration Analysis (JVA) in subjects with bilateral disc displacement with reduction and in subjects with bilateral normal disc position.

**Methods:**

The reliability of selecting the traces was assessed by reading the same traces at an interval of 30 days. The reliability of the vibrations provided by the subjects was assessed by obtaining two tracings from each individual at an interval of 30 min. The validity compared the Joint Vibration Analysis parameters against magnetic resonance imaging as the reference standard. The data were analyzed with exploratory factor analysis.

**Results:**

The short- term reliability of the Joint Vibration Analysis outcome variables showed excellent results. Implementing factor analysis and a receiver operating characteristic as analytical methods showed that six items of the Joint Vibration Analysis outcome variables could be scaled and normalized to a composite score which presented acceptable levels of sensitivity and specificity with a receiver operating characteristic of 0.8.

**Conclusion:**

This study demonstrated that the composite score generated from the Joint Vibration Analysis variables could discriminate between subjects with bilateral normal versus bilateral displaced discs.

**Electronic supplementary material:**

The online version of this article (doi:10.1186/s12903-017-0346-9) contains supplementary material, which is available to authorized users.

## Background

Temporomandibular disorders (TMD) encompass a group of musculoskeletal and neuromuscular conditions that involve the TMJ, the masticatory muscles and all associated tissues; the major symptoms are pain which is often localized in the muscles of mastication or pre-auricular area; joint noises, and limitation in jaw function may be present as additional complaints [1]. Based on the current Diagnostic Criteria, TMD can be classified into three major groups: pain-related; intra-articular; and degenerative joint disease and subluxation disorders [2]. Within the intra-articular group, disc displacement with reduction defines a subgroup in which diagnosis has often been based on clinical finding of joint sounds [3]. Several studies have concluded that TMJ sounds are highly variable [3–5]. Thus, the use of joint sounds as a diagnostic parameter has been questioned [6]. The reliability among calibrated examiners of such sounds has been reported to have a Kappa value of 0.63 [7]. The correct identification of intra-articular conditions using joint sounds has shown a sensitivity of 0.38 and specificity of 0.88, using the Magnetic Resonance Imaging (MRI) as the reference standard [8, 9].

Joint vibration analysis is based on principles of motion and friction by surfaces, which can be captured by accelerometers. Human joints in proper biomechanical relationship, in theory, should produce little friction and little vibration [10–14]; surface changes within the joint could cause greater friction and greater vibration. It has been postulated that different disorders can produce different vibration patterns or signatures in joint including the TMJs [15–17]. Vibration analysis of the TMJ is thus a quantitative process that measures the absolute intensity and frequency distribution of vibratory waves emanating from the joint during jaw motion.

Since there is controversy regarding the utilization of joint vibrations to characterize joint status and consequently diagnosis as presented in a recent systematic review [18], the diagnostic validity of such instrumentation used to measure and characterize this phenomenon must be tested using research designs with strong foundations including reliability evidence, blinded examiners, an acceptable reference standard such as MRI, and acceptable psychometric properties.

Furthermore the progression of joint status in participants with displaced discs has been controversial. While one report postulates a progression from disc displacement to osteoarthritis [19] there is substantial clinical evidence that most untreated patients improve and do not progress over time [20–25]. In addition there is MRI evidence that no change occurs in disc displacement over 22–80 months [26]. More recently the authors of a prospective study that assessed the stability of the temporomandibular joint in disc displacement using MRIs, found that over 8 years of follow-up, 76% of the 789 baseline joint-specific soft-tissue diagnoses did not change [27].

A systematic review [18] reported several limitations in previous reports: (1) lack of blinndness, (2) nonvalidated classification systems, (3) different imaging techniques to identify control and test groups, (4) use of joint sounds per se as evidence of pathology or as a reference standard, and (5) use of joint vibration analysis (JVA) as the reference standard even though it was the device investigated.

The premise of this research was that a more technically accurate instrument and more sophisticated analysis by using factor analysis to select variables might provide more accurate information, compared to auscultation, and more inexpensive, compared to MRI, to assess the phenomenon of joint sounds. Therefore the assessment of vibrations using instrumentation such as Joint Vibration Analysis (JVA) could have the potential to provide data that could be used to assess the phenomenon and to indicate the status of the joint. We focused on the BioJVA produced by BioResearch Associates.

The objectives of this research were, first, to determine if the data associated with joint vibrations could be selected and recorded reliably, second, to analyze the multiple correlated variables with factor analysis to determine if a smaller number of variables could represent the data, and third, to determine if the sensitivity and specificity as represented by the area under the receiver operating characteristic curve were sufficiently large for potential clinical use.

The overall goal of this research was to test the diagnostic validity of the joint vibration output variables against the reference standard of the MRI evaluation by a calibrated radiologist. The underlying analytical strategy was to examine the data with exploratory factor analysis to see (1) how many latent variables, that is, factors, were in the data, (2) whether these latent factors could be interpreted in a reasonable way, and (3) whether a composite score based on the items that survived into the interpretation could be merged into a composite score with adequate sensitivity and specificity as described by a receiver operating characteristic [28, 29].

## Methods

### Subjects

Thirty-six subjects who had undergone an MRI for their TMJs within the last two years agreed to participate in the study. Characterization of bilateral disc displacement or bilateral normal disc position was provided by a calibrated radiologist [8] based on MRI interpretation. The study was approved by the University at Buffalo’s Health Sciences Institutional Review Board and each subject gave informed consent.

### Equipment

The joint vibration analysis (JVA) in the BioPAK^©^ system [30] was leased from Bioresearch Corporation and consisted of a headset encompassing accelerometers on each side, an amplifier, and software for a computer. The signals from the accelerometers were amplified by the small amplifier, which was placed around the subjects’ neck. The amplified signals were transmitted to a PC computer where they were recorded and later analyzed with the software program. Each accelerometer consists of a metal case containing a piezoelectric crystal that has a mass resting on it. This crystal reacts to acceleration by producing a minute electric charge due to compression produced by the mass, which is directly proportional to the acceleration. This is then put into an amplifier of high input impedance prior to being recorded as a vibration signal.

### JVA protocol

The subjects sat in an upright position. Their maximum unassisted opening and lateral deflections were recorded clinically and entered into the computer with the BioPAK software program. The headset device was then placed on the subject’s head with the sensors positioned over the TMJs; the subjects were instructed to watch the monitor where they observed an animation illustrating opening and closing mouth movement, synchronized to a metronome. They were then instructed to open their mouth as wide as they could and close, tapping their teeth together following and matching the animation and the metronome, which they observed on the screen. As the subject performed the opening and closing with the JVA the characteristic vibrations produced by the condyles were detected by the accelerometers and recorded in the computer.

After the first set of JVA tracings were recorded the Research Diagnostic Criteria examination [31] was performed, then a second set of JVA tracings were recorded. The interval between the two sets of tracings was about 30 min.

The variables [4, 30, 32] obtained were: Total Integral I (T), representing a measure of the total amount of energy in the vibration; Integral <300 Hz which is the amount of energy in the vibration that is below 300 Hz; Integral >300 Hz which is the amount of energy in the vibration that is above 300 Hz; >300/<300 Ratio which is the ratio of the high-frequency to low-frequency energy; Peak Amplitude which indicates the highest intensity of the vibration; Peak Frequency which is the frequency at which the highest intensity of the vibration occurred and Median Frequency which is the frequency such that half of the energy is below it and half is above it. These data are provided in an Additional file 1.

### Reliability

Reliability was assessed in three ways. First, the reliability of the examiners for range of motion data between the two clinical examiners was assessed by computing the intraclass correlation coefficient while lateral deviation was assessed with kappa. Second, the ability of two clinicians as a consensus to select the same traces after a three-month interval was assessed by comparing the JVA variables from those two sets of traces. Third, a test-retest protocol assessed the ability of the subjects to provide consistent data by comparing data obtained at a 30-min interval. The examiners were calibrated for Research Diagnostic Criteria for TMD [33].

### Reliability of range of motion assessment

The two clinical examiners in this study were blinded to the MRI diagnosis avoiding bias to the instrumentation under evaluation; examiners were RDC/TMD calibrated [31] and provided the clinical parameters such as range of motion and deflection, which were required by the BioPAK software program to analyze the joint vibrations.

### Reliability of trace selection

The selection of vibration data was done as a consensus by the two clinical examiners following the manufacturer’s protocol, which included the selection of the five largest vibration amplitudes in a trace of six to eight open-close cycles. To determine if the examiners could reliably select the JVA traces with the largest time-based signals, 15 traces were randomly selected and the traces were read at two times 3 months apart.

The recorded JVA traces were then analyed for the largest vibration amplitude that consistently occurred in each joint recording from the JVA sweep. Five large vibration amplitudes were selected from each trace by both examiners and were used to calculate frequency spectrum computed by the Fast Fourier Transform algorithm. These spectra were used for this estimation of reliability.

### Test-retest reliability

Data from the first recordings from the subject were compared with the recordings made 30 min later.

### Factor analysis

The number of latent variables underlying the data was investigated by exploratory factor analysis. The (log) data for each item was scaled by subtracting its mean and dividing by its standard deviation to form a z-score. The mean across the six items was taken as a composite score for each subject and a receiver operating curve was generated.

**Fig. 1:**
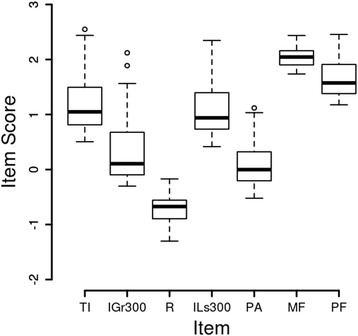
Box Plot of JVA Data. The item score is the logarithm base 10 of the original data. These data, based on all 36 subjects, largely corrected the skewed distributions. The items are TI: total integral, IGr300: integral greater than 300 Hz, R: ratio of integral >300 to integral <300, ILs300: integral less than 300 Hz, PA: peak amplitude, MF: median frequency, PF: peak frequency

Factor analysis seeks to condense a larger number of correlated variables into a smaller number of underlying, interpretable variables which explain the bulk of the relationships among the original variables. Graphical inspection of the raw data suggested that they were strongly non-Gaussian so the logarithm to the base 10 was taken of each data point. The box plot based on these logarithms suggested a better distribution for each variable (Fig. 1) and Shapiro-Wilks tests of each item within each group supported this. Due to doubts about the independence of data from the right and left joints [34], only the data from the right side were analyzed. Statistical Analysis

Reliability of dichotomous variables was assessed by percent agreement and Kappa values. Reliability of continuous variables was assessed by intraclass correlation coefficients. Validity was assessed by calculating the Receiver Operating Characteristic (ROC) using the composite score identified with the factor analysis.

The data were analysed using the R statistical and graphics package [35].

## Results

### Demographics

A total of 36 subjects (21 females, 15 males) participated in this study. The mean age was 39.03 ± 13.6 years. There were no statistically significant differences in age by gender. Twenty-one subjects (11 males and 10 females) had normal bilateral joints and 15 subjects (4 males and 11 females) had bilateral joints with disc displacement with reduction.

### Reliability of range of motion assessment

The ability of the two clinicians to obtain reliable clinical data led to excellent intraclass correlation coefficients (ICC) as shown in Table 1.

**Table 1:** Reliability estimates

Item	ICC or Kappa	95% CI*
Clinical Data		
Pain Free Opening	0.94	0.67 – 0.99
Maximum Unassisted Opening	0.88	0.40 – 0.98
Maximum Assisted Opening	0.98	0.89 – 1.0
Lateral Deviation	87.5% (Kappa)	
Test-Retest Reliability of Selection of JVA Traces		
Total Integral	0.99	0.98 – 1.0
Integral <300	0.99	0.97 – 0.99
Integral > 300	1.0	0.99 – 1.0
> 300/< 300 Ratio	0.91	0.82 – 0.96
Test-Retest Reliability of JVA traces provided by subject at 30 min		
Total Integral	0.90	0.84 – 0.93
Integral <300	0.89	0.83 – 0.93
Integral > 300	0.91	0.87 – 0.94
Ratio >300/< 300	0.63	0.44 – 0.76
Peak Amplitude	0.87	0.81 – 0.92
Peak Frequency	0.77	0.65 – 0.85
Median Frequency	0.70	0.54 – 0.80

*None of the confidence intervals include zero so all p’s are less than 0.05

The ICCs from traces selected at the three months interval by consensus of the two clinical examiners also showed excellent values (Table 1). These data suggest that the examiners reliably identified the joint vibrations in the traces three months apart and therefore the results obtained at the validity stage would not be biased by the ability of the examiners to identify the phenomenon using the instrumentation.

### Reliability of joint vibrations by test-retest

The reliability of the joint vibrations as a phenomenon was evaluated over a period of 30 min. The ICCs for the JVA variables showed excellent values except for the Ratio >300/<300 item (Table 1). Based on the excellent reliability results, the mean between trace 1 and trace 2 was taken as the outcome variable for further analysis.

### Factor analysis

Graphical inspection of the raw data suggested that they were strongly non-Gaussian so the logarithm to the base 10 was taken of each data point. The box plot based on these logarithms suggested a better distribution for each variable (Fig. 1) and Shapiro-Wilks tests of each item within each group supported this. Due to doubts about the independence of data from the right and left joints, only the data from the right side were analysed.

The Cronbach's Alpha was 0.90 with 95% confidence limits from 0.82 to 0.98. The Kaiser-Meyer-Olkin measure of sampling adequacy was 0.74 for the overall data set and the minimum item was 0.42 for the “Ratio” item. The next lowest measure of sampling adequacy (MSA) was 0.71 for the “Median Frequency” item. Notice (Table 2) that for the ratio the loading is low and the communality is low. For these reasons (supported by the low reliability in Table 1), the ratio was deleted from the subsequent analysis.

**Table 2:** Data from exploratory factor analysis

Item	Pattern loadings	Communality	Mean	Std Dev
Total Integral	0.97	0.94	1.231	0.534
Integral > 300	0.98	0.97	0.393	0.596
Ratio	0.49	0.24	−0.752	0.260
Integral <300	0.95	0.90	1.152	0.526
Peak Amplitude	0.87	0.76	0.167	0.462
Median Freq. Frequency	0.73	0.53	2.021	0.176
Peak	0.69	0.48	1.626	0.300

A scree plot (not shown) suggested that one or two factors might be allowed. Two factors led to several cross-loadings in the pattern matrix coefficients, some communalities greater than one, and no interpretation, so one factor was used.

The pattern loadings, communalities, means, and standard deviations for each item are shown in Table 2. A box plot of all seven items is shown in Fig. 1.

**Fig. 2:**
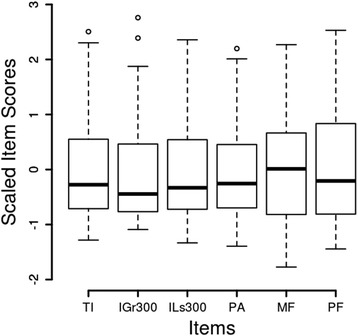
Box Plot of Scaled JVA Data. In order to form a composite variable the data for each item were normalized by subtracting the mean and dividing by the standard deviation

**Fig. 3:**
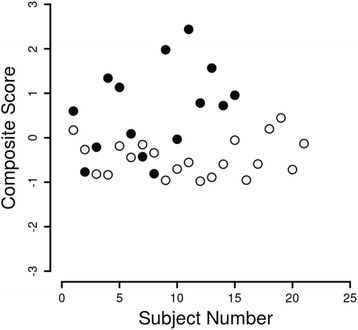
Composite Scores. The filled circles are composite scores of the subjects with bilateral disc displacements and the open circles are the composite scores of the subjects with bilateral normal discs

Each of the six items that were kept was scaled to a mean of zero and a standard deviation of one, a z-score (Fig. 2). The mean across the six scaled items was taken as a composite score for each subject. A plot (Fig. 3) of the composite score for each subject in the disc displacement and normal groups suggested the scores differed.

**Fig. 4:**
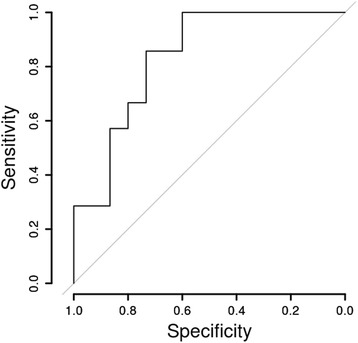
Receiver Operating Characteristic. The composite scores in Fig. 3 were used to generate the Receiver Operating Characteristic curve where the subjects with bilateral normal discs were used for specificity and the subjects with bilateral displaced discs were used for sensitivity. The area under the curve is about 0.82

The composite scores from the subjects in the two groups (Table 3) were used to generate a receiver operating curve where the scores from the subjects with bilateral disc displacement was used for the sensitivity and the scores from the subjects with bilateral normal discs were used for the specificity (Fig. 4). The area under the receiver operating characteristic curve was 0.82. A composite score of −0.04 led to a sensitivity of 0.86 and a specificity of 0.73, this score is the closest to the ideal score of 1.0 for sensitivity and 1.0 for specificity. A composite score of −0.24 led to a sensitivity of 0.67 and a specificity of 0.80, this score is the closest to the sensitivity of 0.70 and specificity of 0.95 suggested by Dworkin and LeResche ([31], pp. 318–319).

**Table 3:** Composite scores of all participants by group

Normal	DDwR
0.171	0.600
−0.266	−0.769
−0.818	−0.212
−0.833	1.339
−0.185	1.133
−0.443	0.087
−0.154	−0.428
−0.341	−0.809
−0.957	1.978
−0.703	−0.033
−0.555	2.437
−0.977	0.780
−0.889	1.566
−0.595	0.723
−0.054	0.956
−0.953	
−0.591	
0.199	
0.444	
−0.716	
−0.131	
0.600	
−0.769	
−0.212	
1.339	
1.133	
0.087	
−0.428	
−0.809	
1.978	
−0.033	
2.437	
0.780	
1.566	
0.723	
0.956

## Discussion

The main findings of this research were that clinicians can reliably identify the tracings generated by the JVA instrumentation, that the vibration generated by the TMJs are reliable within a 30 min time period, that the data have good psychometric properties, and that exploratory factor analysis led to a composite score which had a good receiver operating characteristic.

The strengths of this research include clinical diagnostic criteria with trained and calibrated examiners, MRI TMJ soft tissue characterization by a calibrated radiologist who was blinded assessment of the data, and the use of exploratory factor analysis to assess the properties of the data and to retain relevant items from the vibration instrumentation.

We recognize that there are limitations associated with this investigation. First, this sample only included individuals with either bilateral disc displacements with reduction or bilateral normal position of the disc. This design made the results easier to interpret since there are reports that the vibration from the TMJ on one side might be detected on the other side [34, 36–38]. Clearly, unilateral disc displacement subjects need to be studied in the future, as well individuals with other intra-articular conditions in order to have a better representation of the TMD population.

Second, the imaging data and the vibration data were not concurrent. While some reports postulate a model of progression from disc displacement to osteoarthritis there are four lines of evidence that argue against the progression model. First, a large, recent, cross-sectional study failed to find evidence in favor of progression [26]. Second, several longitudinal clinical studies failed to find evidence of progression [20–25]. Third, a study with pre and post MRI imaging failed to find evidence of progression [26]. And fourth, a prospective 8 year follow-up study found that 76% of the joint diagnoses were stable [27] Therefore, although it would be preferable to have a fully parallel data set for the imaging and vibration assessment, there is no evidence that the current design jeopardized the results.

Our reliability results confirmed recent results [39], extended the short-term reliability from 3 min to 30 min, and extended the study population from healthy participants to a group of individuals with bilateral disc displacement with reduction.

While we believe that our approach is innovative, we want to clearly express that in its current format the approach is not ready for clinical diagnostic application. Future research could lead to the potential utility for characterization of disc position of the TMJs and to better understand the potential role of such vibrations in the intracapsular TMJ status and its impact in jaw function.

## Conclusions

The excellent reliability obtained by the examiners reading the JVA data demonstrates that examiners can be properly trained and they can reliably identify and interpret the pertinent data produced by this technological device. In addition, the assessment of the joint vibration as phenomena can be reliably assessed within a short period of time.

Using a six-item composite score a receiver operating curve was generated (value of 0.82) suggesting that this composite score based on the vibration characterization can be used to discriminate between normal disc position and displaced disc position.

Nevertheless, the authors would like to emphasize that the results must be interpreted with caution due to the fact that the composite score is not generated by the instrumentation software, the independence of signals from each TMJ is not yet established, and because the study sample does not represent the entire spectrum of disc displacements and degenerative joint disease.

## Additional file

Additional file 1: An additional file named “JVAdata.csv” contains the MRI-based group and the JVA-based data. (CSV 1 kb)

## Abbreviations

ICC: Intraclass correlation coefficient
JVA: Joint vibration analysis
MRI: Vmagnetic resonance imaging
TMD: Temporomandibular disorders
TMJ: Temporomandibular joint
